# Sport Activity for Health!! The Effects of Karate Participants’ Involvement, Perceived Value, and Leisure Benefits on Recommendation Intention

**DOI:** 10.3390/ijerph15050953

**Published:** 2018-05-10

**Authors:** Ying-Chih Chang, Tsu-Ming Yeh, Fan-Yun Pai, Tai-Peng Huang

**Affiliations:** 1 Department of Leisure and Recreation Management, Da-Yeh University, ChangHua 515, Taiwan; n1208940@mail.dyu.edu.tw (Y.-C.C.); htp0719@yahoo.com.tw (T.-P.H.); 2 Department of Industrial Engineering and Management, National Quemoy University, Kimen 892, Taiwan; 3 Department of Business Administration, National Changhua University of Education, ChangHua 500, Taiwan

**Keywords:** involvement, perceived value, leisure benefits, recommendation intention

## Abstract

This study intends to discuss the effects of participants’ involvement, perceived value, and leisure benefits on recommendation intention in the sport of karate. The questionnaires were collected online by karate clubs on Facebook and included 369 valid participants. The research findings show that karate participants from different places of residence do not display significant differences in involvement, perceived value, leisure benefits, and recommendation intention. Furthermore, “attraction” in the involvement category reveals the highest mean, “paid spirit and energy being worthy” in perceived value appears as the highest mean, and “physiological benefits” in leisure benefits shows the highest mean. The Pearson correlation analysis result presents significant strong positive correlations between involvement, perceived value, leisure benefits, and recommendation intention. Finally, multiple regression analysis reveals that leisure benefits, except “physiological benefits”, show notably positive effects on recommendation intention. According to the research results, suggestions are proposed for the reference of karate teaching business managers, participants, and future research.

## 1. Introduction

Leisure activities are beneficial for health [1]. They can help maintain physical and mental health by acting as a buffer to stress and can also help people recover from stress [2,3]. Some of the main benefits of leisure activities are social. Doing such activities with other people can provide social support [4]. Since leisure activities are often done in groups, they provide a great opportunity to make new relationships and strengthen existing ones [5].

Karate is a martial art based on the skills of strikes, punches, and kicks [6]. It can activate blood circulation, increase muscle endurance, enhance vital capacity, and promote reaction capability without specific places or special props, but an adequate space for practice, that it is regarded as the most economical of sports. Furthermore, karate is not simply exercise in groups, as it can also be practiced individually. It is considered as the recreation sport most suitable for individuals in modern life. From the viewpoints of personal, physical, and mental health and the development trend of recreation sports, karate is a recreation sport suitable for the citizens and worthy of promotion [7].

Word of mouth has great influence and is persuasive among consumers [8]. Positive word-of-mouth recommendations not only could attract new customers to increase the revenue of an enterprise but could also reduce marketing expenditures [9]. With the changing recreation sports industry, there is an urgent concern among karate teaching business managers to enhance existing trainees’ recommendation intention in order to continuously increase the number of new participants in a fiercely competitive environment. Involvement, referring to perceived relevance based on personal needs, interests, and value are critical to discuss when considering consumer behaviors in the leisure domain [10]. Besides, perceived value is an important antecedent to predict the future behavioral intention of consumers or activity participants [11]. International research on sports industry participants also supports such a theory, that participants’ perceived value of products or events is a key factor in repurchase intention and reparticipation [12]. The benefits acquired from participating in leisure activities are called leisure benefits. Mannell and Stynes [13] discovered that leisure participants would be stimulated by time, environment, activity, and mood to generate psychological, physiological, environmental, economic, and social influence, which would become leisure benefits through personal subjective judgment. Ajzen [14] regarded leisure benefits as an individual achieving the goal of participating in leisure. In this case, leisure benefits are positive and beneficial changes after an individual participates in leisure activities. The acquisition of leisure benefits is an important consideration for an individual participating in a leisure activity as well as a key factor in participants thinking over the subsequent behaviors [3].

In sum, this study expects to understand the effects of involvement, perceived value, and leisure benefits on karate participants’ recommendation intention and propose conclusions and suggestions, according to the research results, for the reference of relevant parties.

## 2. Literature Review

### 2.1. Involvement

Zaichkowsky [10] explained involvement as individual concern about certain things and classified the factors in personal involvement into three categories: (1) personal factors, containing interests, value, or needs, would facilitate a person moving towards the goal because of certain motivation; (2) physical factors referred to different personal concerns caused by the characteristics of things; and (3) situational factors referred to the factors in temporarily appearing correlations or interests in target objects because of special situations. Celsi and Olson [15] defined involvement as the personal perception of things and individual relevance under specific time and situations. Rothschild [16] argued that involvement was induced by special situations or stimuli, was a kind of invisible motivation or concern, and would affect data collection and decision making. Early concepts linked involvement to the description or prediction of consumer behaviors and regarded involvement as a variable of individual consumption [17].

McIntyre [18] mentioned the meaning of involvement in the sports leisure domain as participants in recreation sports acquiring pleasure and meaning in the process who would gradually become involved in sports leisure activity. Kyle and Mowen [19] indicated that an individual would enhance the motivation to participate in leisure activity when perceiving the value of that specific leisure activity as being able to enhance living benefits of stress relief and physical health promotion. Mannell and Iso-Ahola [20] proposed that the key factors of internal leisure motivation and inner happiness perception of leisure lie in maintaining or developing favorable leisure activity interests. The promotion of leisure involvement allows for an individual to acquire benefits from leisure and to perceive pleasure from the engagement in leisure [21]. Havitz and Dimanche [22] further transformed involvement from consumers’ behavioral roles into personal opinions about leisure activity and indicated that involvement was a personal psychological state about leisure activities which could drive behaviors after being induced by situations or stimuli. Consequently, an individual would enhance involvement in leisure after perceiving the value of leisure as being able to promote living benefits. Further, the promotion of leisure involvement allows individuals to acquire benefits from leisure and to perceive the joy of leisure.

Aiming at the sports leisure domain, Havitz and Dimanche [22] defined involvement as unaware awakening or interests between an individual and sports leisure. Venkatraman [23] defined continuous involvement in sports as being enthusiastic about physical activity with strong interest and regarding sports as a hobby. Kim et al. [24] divided leisure involvement into behavior involvement and sociopsychological involvement. Behavior involvement referred to an individual investing energy and time in a specific activity as the explicit behaviors. Sociopsychological involvement, as the state between an individual and the sports leisure activity, was the pleasant perception and self-performance achieved through activity [25]. In the research on tennis players, Wu and Wu [26] regarded leisure involvement as perceived interests, stimuli, or an awakening state. Cheng and Chang [27] pointed out involvement as the degree of sports participants engaging in sports who would generate subsequent concerns with deepening involvement.

Research on involvement in recreation sports mainly focuses on the sociopsychological level. Some researchers would supplement behavior involvement according to different leisure behaviors. Involvement is an unaware awakening or interested psychological state as well as a personal idea and attitude towards the engagement in recreation sports [1]. Accordingly, karate participants’ involvement is defined in this study as karate participants’ engagement in karate sports and the inner emphases on and interests in karate, from which the importance and influence of karate sports on individuals are apparent.

### 2.2. Perceived Value

Monroe and Krishnan [28] proposed that consumers would present positive perceived value of a product when the received perceived quality was higher than the perceived sacrifice. In the transaction utility theory, Thaler [29] also mentioned that perceived value was the reference for consumers considering the purchase. In the experimental research on consumers, Dodds and Monroe [30] pointed out the correlation between product price, product quality, and perceived value. Apparently, research on perceived value at the time referred to the difference between physical products and product price.

Zeithaml [31] concluded four points of view about customers’ perceived value. (1) Value was a lower price. Customers stressed mostly on prices that products with same functions would present higher value on lower prices. (2) Value was customers’ subjective desire for products. Value was the degree of customers acquiring satisfaction with personal needs. (3) Value was a trade-off between paid prices and received quality. Value was a trade-off between paid money and acquired products. (4) Value was to acquire the paid-for product or service. In consideration of all received and given elements, value was consumers acquiring the paid-for product or service. Dodds et al. [32] indicated that consumers would preset product prices before purchase. When finding out a lower product price than the set price, consumers would reveal a positive perception; perceived value was the net profit after the purchase. Gale [33] pointed out perceived value as consumer perception after comparing products or services among competitors in the market; that is, it was a comparison idea. Tam [34] regarded it as the result of consumers evaluating received services and paid costs. Sirdeshmukh et al. [35] considered that perceived value was the difference between paid costs and received profits when consumers maintained a relationship with service providers. As a consequence, the idea of perceived value, as consumers’ subjective perception, covers tangible products as well as intangible services.

Aaker [36] discovered that customers’ perceived value of prices was the core of perceived value. Fandos et al. [37] stated that the key characteristics of customers’ perceived value were perceived by customers, but not objectively determined by sellers; therefore, only customers could perceive the value of products or services offered by sellers. Perceived value is consumers’ subjective perception of products or services. Consumers might continuously purchase products or services with higher perceived value, which might even affect the subsequent behavioral intention, including recommendation to others. Enterprises therefore have to enhance consumers’ perceived value of products or services. However, there would eventually appear differences in perceived value due to customers’ psychological conditions. Consequently, perceived value is defined in this study as karate participants perceiving the paid money, time, energy, and spirit being worthy after participating in karate exercise to further recommend to others.

### 2.3. Leisure Benefits

Benefits, as an advantage, refer to positive changes in the individual, society, economy, environment, or other dimensions [2,38]. Bammel and Burrus-Bammel [39] regarded leisure benefits as individuals receiving advantages from participating in leisure activity. In this case, leisure benefits could be an individual acquiring certain positive improvements from leisure activity. Mannell and Kleiber [40] pointed out the effectiveness of leisure benefits to help an individual maintain certain standards so as to avoid having a low spirit or sense meaningless life when not participating in leisure activity. Iwasaki [41] announced in an evaluation of leisure benefits that leisure allows human beings in the world to acquire a valuable and meaningful life and could help people promote their quality of life. For this reason, the benefits acquired from an individual participating in leisure activities could basically keep individual life efficiency on a certain level to further receive positive improvements in life.

Coleman [42] considered that the interpersonal interaction among friends, neighbors, and family members through leisure activity could help each other maintain psychological health, generate psychological health, and further enhance happiness perception in life. Siegenthaler [43] found leisure to be the critical buffer in negative living events, which could help reduce negative influences on an individual. Fenech [44] also indicated that freely choosing leisure activity for the sense of achievement could help an individual regulate the stress in life, generate self-identification, and find out self-value. By integrating the viewpoints of Coleman [42], it is found that engaging in recreational leisure activity with relatives and friends could help an individual reduce the impact of negative events in life, enhance spiritual, emotional, and psychological health, and generate psychological health.

Wankel and Berger [45] pointed out leisure benefits as the achievement of goals and considered that an individual being able to achieve a goal through leisure activity was the key to evaluating leisure benefits. Ajzen [14] also agreed that leisure benefits were the achievement of leisure goals, including the achievement of leisure participation goals and the belief of participants in leisure being able to help them achieve the set goals. Consequently, when an individual anticipates high profits from participating in a leisure activity, the participation intention would be increased to perform higher activeness for the activity.

Driver et al. [38] regarded benefits as an advantage and explained it as a gain, and that leisure benefits were the subjectively perceived improvements after participating in a leisure activity. Mannell and Stynes [13] constructed a leisure benefit system and indicated that participants in leisure activities would generate psychological, physiological, economic, environmental, and social influence when participating in such activities because of external stimuli of environment, activity, time, and mood. Such an influence would present leisure benefits after individual evaluation. Summing up the above opinions, distinct leisure activities will generate different leisure benefits for individuals. An individual might merely generate physiological and psychological benefits when being alone, but social improvement might be perceived during group activity leisure [3]. Karate allows independent practice and group exercise so that karate participants’ leisure benefits are therefore defined in this study as the subjectively perceived positive psychological, physiological, and social improvement of an individual after participating in karate.

### 2.4. Recommendation Intention

Arndt [46] regarded word of mouth as the oral and direct communication among people about real brands, products, or certain services. When discussing the effect of consumers’ word of mouth on judging the preference for products, Bone [47] defined word of mouth as the communication among people where any party participating in the communication was not the marketing source. Anderson [48] mentioned that word of mouth covered positive and negative propagation and was a neutral term. Blackwell et al. [49] defined word of mouth as people delivering comments, ideas, or information through informal channels. Harrison-Walker [50] regarded word of mouth as an overall comment aimed at companies, services, or specific products flowing among people. The rapid growth of the Internet has provided online platforms that have become a popular channel for consumers sharing experiences and expressing opinions [51]. Mobile media tools provide real-time experience sharing and rapid information delivery interface [52,53,54]. In sum, word-of-mouth recommendation is the positive comments aimed at a unit organization or brand, products, or services delivered among consumers by use of various informal channels without business intention.

Day [55] discovered that a consumer might change the neutral or even negative attitudes into positive attitudes through others’ word of mouth, which could achieve nine times that of advertising performance. Nevertheless, the reliability of word of mouth would be enhanced when the it was not for commercial use but was from someone who was emphasized or trusted by consumers [56]. As a consequence, word of mouth has been considered as a reliable and trustable information source. Further, the effect of networks has maximized the influence of word of mouth. Research on word of mouth therefore has been emphasized in academia and in practice [57].

Herr et al. [58] mentioned that word of mouth was often studied in correlation to marketing. Mahon [9] indicated that the positive recommendation of products not only could attract new customers to effectively increase the revenue of enterprises but could also reduce the marketing expenditures of enterprises. Besides, positive recommendation could help enterprises build a favorable public image. Fullerton [59] supported the claim that positive word of mouth of consumers could create new profits for businesses. A business not being able to retain old customers would increase the cost burden of the business owner so that it would spend more on searching for new customers, in addition to losing original customer profits. As a result, customers’ positive recommendation intention could assist enterprises in increasing the revenue from new customers as well as helping enterprises reduce costs. Parasuraman et al. [60] pointed out word-of-mouth intention, recommendation to others, and complaint possibility as the measurement of customer loyalty. Ones who were willing to establish positive word of mouth for an enterprise were loyal customers [61,62]. Recommendation intention is therefore defined as a primary indicator in measuring customer loyalty [63].

Word of mouth contains positive and negative propagation and is a neutral term. Positive word-of-mouth propagation of karate participants could help karate teaching business managers strive for potential participants and create new profits. As a result, it is an important marketing strategy to promote participants’ recommendation intention. Recommendation intention is defined in this study as the intention of participants being willing to deliver positive word of mouth about karate through oral, network, or other communication channels after experiencing karate practice.

### 2.5. Research Hypotheses

Cronin et al. [64] indicated that consumers’ positive behavioral intentions contained the willingness to describe the advantages of a company, recommendations to others, showing the company loyalty, increasing the purchase frequency, and paying higher prices. Chung et al. [65], Gronholdt et al. [66], and Kuo et al. [67] depicted behavioral intention to include recommendations to others and revealed that involvement would positively affect behavioral intention. Consumers’ recommendation behaviors therefore could be one of indices of positive behavioral intention.

Yang et al. [68] proposed that higher involvement leads to higher satisfaction. Higher satisfaction always leads to positive behavioral intention. For karate participants, having more knowledge about karate, and thus he or she might be more satisfied with karate exercise. More satisfied participants are more likely to recommend to others that they should practice karate. Therefore, the first hypothesis is derived in this study.

**Hypothesis** **1.** *Karate participants’ involvement positively affects the recommendation intention.*

Perceived value presents positive effects on subsequent behaviors [69,70]. In this case, customers would enhance the willingness of subsequent behaviors when perceiving certain things with high value. Yang et al. [71], Chuang et al. [72], and Dowling and Hammond [73] measured behavioral intention with recommendations to others, reparticipation intention, and game sharing and discovered that sports participants’ perceived value would positively affect behavioral intention. Chuang et al. [72] presented behavioral intention with propagation to others, recommendation to others, encouraging others, and continuous participation. Chuang et al. [72] also found out the positive effects of perceived value on visitors’ behavioral intention. The results in Kim et al. [74] illustrated that higher perceived value leads to more satisfaction and then results in higher recommendation intention. Based on previous researches, Hypothesis 2 is proposed as follows.

**Hypothesis** **2.** *Karate participants’ perceived value positively affects the recommendation intention.*

Individuals perceive stimuli after participating in leisure activities. The stimuli create psychological, physical, and social effects. After evaluating the effects, individuals can perceive benefits [75,76]. Wang [77] measured after-visit behaviors with word-of-mouth recommendation and the analysis results showed that recreation benefits had significantly positive effects on recommendation intention.

In addition, Pi et al. [78] measured leisure benefits with physiological, psychological, and social benefits and measured loyalty with behavioral intention and recommendation to others after participating in a hiking activity. The results of Pi et al. [78] showed that leisure benefits presented remarkable predictive power on recommendation intention and the better the leisure benefits, the higher the loyalty. According to above literatures, the third hypothesis is proposed in this study.

**Hypothesis** **3.** *Karate participants’ leisure benefits positively affect the recommendation intention.*

## 3. Materials and Methods

### 3.1. Research Hypothesis and Framework

This study aims to understand the effect of karate participants’ involvement, perceived value, and leisure benefits on recommendation intention and proposes the following hypotheses based on the research objective.

**Hypothesis** **4.** *Karate participants’ involvement positively affects the recommendation intention.*

**Hypothesis** **5.** *Karate participants’ perceived value positively affects the recommendation intention.*

**Hypothesis** **6.** *Karate participants’ leisure benefits positively affects the recommendation intention.*

**Figure 1:**
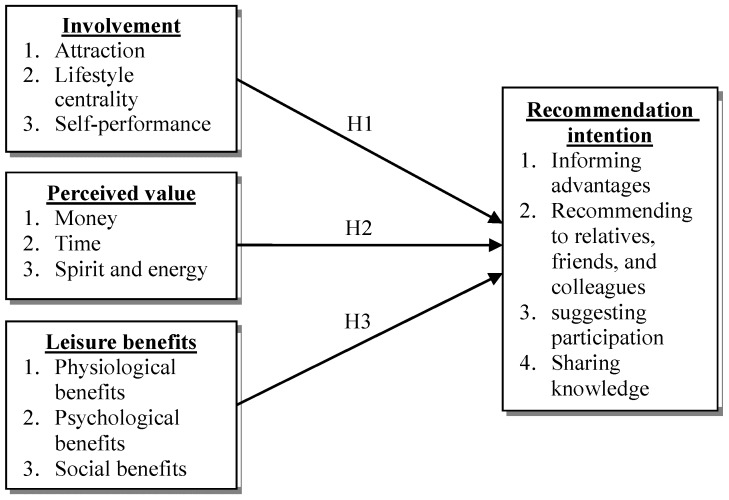
Research framework.

According to literatures and the research hypotheses, the research framework is shown in Figure 1.

### 3.2. Research Instrument

A questionnaire survey was utilized in this study. The questionnaire contained five parts: (1) involvement scale; (2) perceived value scale; (3) leisure benefits scale; (4) recommendation intention scale; and (5) personal basic data investigation. The involvement scale, with a total of 15 questions, was revised from the leisure involvement scale proposed by Dai et al. [79] and Chen and Lin [80], including three dimensions of “attraction”, “lifestyle centrality”, and “self-performance”. The perceived value scale contained three questions: (1) I feel that the money spent for participating in karate is worthy; (2) I feel that the time spent participating in karate is worthy; and (3) I feel that the spirit and energy paid for participating in karate is worthy. Referring to Chang-Liao [81], the scale modified the semantics according to the research characteristics. The leisure benefits scale referred to the serious leisure benefits scale proposed by Hsu [82], covering three dimensions of “physiological benefits”, “psychological benefits”, and “social benefits”. Moreover, it also referred to Huang et al. [83] and modified the semantics according to the research characteristics to compile 12 questions suitable for this study. The recommendation intention scale included four questions: (1) I would tell my relatives, friends, and colleagues about the advantages of karate; (2) I would recommend karate to my relatives, friends, and colleagues; (3) I would suggest anyone who intends to participate in martial-arts recreation sports to participate in karate; and (4) I am glad to share the knowledge about my participation in karate. The scale referred to the word-of-mouth recommendation intention scale proposed by Chang et al. [76] and modified the semantics according to the research characteristics, aiming to understand karate participants’ recommendation intention.

Research variables of the scales were scored with Likert’s five-point scale, including: (1) extremely disagree; (2) disagree; (3) slightly agree; (4) agree; and (5) extremely agree, which are orderly given the scores of 1 to 5. The higher scores represent the higher identification of participants. “Personal basic data investigation” contained 11 questions about participants’ gender, age, marital status, educational attainment, occupation, place of residence, personal monthly income, karate participation seniority, and average times of weekly independent practice and group exercise participation in past three months.

### 3.3. Research Subject and Data Collection

Since the actual national population of those participating in karate cannot be surely confirmed, karate clubs on Facebook were utilized for the data collection. From March 30, 2016, the number of national karate club members on Facebook was investigated, and national karate participants aged above 15 were studied during March 30–April 5. With convenience sampling and snowball sampling, online karate clubs were formally distributed the questionnaire for the survey. The researcher first requested the intention of respondents by sending messages to karate clubs on Facebook, offered the questionnaire page, and asked respondents to invite others to join in. As snowball sampling was applied on the Internet, the distributed questionnaires could not be accurately estimated. A total 389 copies of the questionnaire were collected, including 369 valid ones. The retrieval rate was 94.9%.

### 3.4. Statistical Analysis Methods

In this study, descriptive statistics was first used to summarize the respondents’ profiles. Cronbach’s α was calculated for each variable as the estimate of the reliability of a psychometric test [84]. Correlation coefficients were calculated to analyze the correlation between research variables. Finally, regression analyses were employed to verify the hypotheses in our proposed model. We used SPSS version 18 (SPSS, Chicago, IL, USA) to execute all the aforementioned analyses.

## 4. Research Results

### 4.1. Analysis of Sample Basic Data

Descriptive statistical methods were applied to the sample distribution. Most respondents (266) were male, about 72.1% of total samples; most respondents (90) were aged 31–41, about 24.4%; most respondents (221) were single, about 59.9%; most respondents (214) presented the educational attainment of college or university, about 58%; most respondents (116) were students, about 31.4%; most respondents (155) lived in the north, about 42%; most respondents (96) did not have recurring income, about 26%; most respondents (108) showed the karate participation seniority of 4–10 years, about 29.3%; most respondents (125) presented the karate rank of grade 2–3, about 33.9%; most respondents (108) showed an average of 1–2 times of weekly independent practice and group exercise participation in past three months, about 29.3%; and most respondents (155) did not participate in other martial art sports, about 42%. Table 1 shows the analysis of sample basic data.

Furthermore, the description and analysis of the research variables are shown in Table 2. By observing the “involvement” scale, the overall involvement of karate participants appears as the mean at 4.010, presenting high overall involvement of karate participants. In terms of the subdimensions, attraction in karate participants’ involvement reveals the highest mean 4.497, while lifestyle centrality appears as the lowest mean at 3.642. By observing the “perceived value” scale, the mean of overall perceived value is 4.341, showing that karate participants are highly assured of the participation in karate exercise. In regard to the questions “paid spirit and energy being worthy” in karate, participants’ perceived value reveals the highest mean of 4.507, while “spent money being worthy” appears with the lowest mean of 4.146. By observing the “leisure benefits” scale, the mean of overall leisure benefits is 4.284, revealing that karate participants are highly assured of the leisure benefits after participating in karate practice. Regarding the subdimensions, physiological benefits in karate participants’ leisure benefits show the highest mean of 4.498, while social benefits has the lowest mean of 3.887. By observing the “recommendation intention” scale, the mean of overall recommendation intention appears as 4.251, showing that karate participants are highly willing to recommend karate to others. In terms of the questions “I am glad to share my participation in karate” in karate participants’ recommendation intention reveals the highest mean of 4.431, while “I would recommend karate to my relatives, friends, and colleagues” shows the lowest mean of 4.098. It reveals that karate participants are willing to recommend karate to others, but the intention of active recommendation is inadequate.

### 4.2. Reliability and Validity Analysis of Scale

Cronbach’s α was used to evaluate the internal consistency and reliability of each dimension. A higher Cronbach’s α value reflects greater internal consistency among the questionnaire items. With regard to the acceptable level, Nunnally [84] suggests that a Cronbach’s α greater than 0.9 indicates high reliability, 0.7 < α < 0.9 medium reliability, while less than 0.5 reveals low reliability and thus the item should be rejected. The reliability of each dimension was between 0.828 and 0.917, with a Cronbach’s a coefficient of 0.953 for the entire questionnaire scale. These results indicate that the reliability of the questionnaire is good. Table 3 presents the favorable internal consistency.

Validity measures can be divided into content and construct validity. The questionnaire in this study was developed based on theories in literatures, referring to the questionnaire contents proposed by Chang et al. [76], Dai et al. [79], Chen and Lin [80], Chang-Liao [81], Hsu [82], and Huang et al. [83], and was modified after discussing with experts that it shows certain content validity.

### 4.3. Correlation Analysis

After correlation analysis of involvement, perceived value, leisure benefits, and recommendation intention (Table 4), all research variables and dimensions show a positive correlation with recommendation intention, which achieves significance. It reveals that the higher involvement, perceived value, and leisure benefits of the karate participants, the higher the recommendation intention.

### 4.4. Regression Analysis

Regression analysis was utilized to verify the hypotheses. Before regression analysis, chi-squared test was conducted to test the normality for each variable. The results showed that the data of each variable are normally distributed. Forced entry method was chosen to do the regression analysis. For example, we first regressed recommendation intention on involvement. Three dimensions of involvement—attraction, lifestyle centrality, and self-performance—are forced to act as independent variables in the regression model and recommendation intention is the dependent variable. The regression analysis results are shown in Table 5.

In Table 5, VIF < 10 means there was no collinearity between independent variables. The variance inflation factor (VIF) is two collinearity diagnostic factors that can help to identify multicollinearity. The VIF measures the impact of collinearity among the variables in a regression model. Values of VIF that exceed 10 are often regarded as indicating multicollinearity [3]. It was also found that independent variables achieve significance, where attraction appears to be the highest predictive power (β coefficient = 0.330). After the adjustment with multiple regression analysis, *R*^2^ = 0.352 represents the variance explained as 35.2%, and attraction, lifestyle centrality, and self-performance show positive effects on recommendation intention.

Proceeding regression analysis of perceived value and recommendation intention (Table 6), where perceived value is the independent variable and recommendation intention is the dependent variable, the independent variable significantly influences the dependent variable (β coefficient = 0.619). After adjusting with regression analysis, *R*^2^ = 0.300 stands for the variance explained as 30.0% and perceived value appears to have positive effects on recommendation intention.

Regression analysis was applied to the subdimensions of leisure benefits and recommendation intention (Table 7), where physiological benefits, psychological benefits, and social benefits are independent variables and recommendation intention is the dependent variable. First, VIF < 10 represents no collinearity between independent variables. Second, the analysis result shows that merely physiological benefits do not achieve significance, and psychological benefits among subdimensions achieve significance, showing higher predictive power (β coefficient = 0.401). After adjusting with multiple regression analysis, *R*^2^ = 0.395 stands for the variance explained as 39.5%, and psychological benefits and social benefits present positive effects on recommendation intention.

From above analyses, the test results of research hypotheses are organized in Table 8.

## 5. Discussion

According to the research results, karate participants’ involvement has significantly positive effects on recommendation intention. Such a result conforms to the past research on involvement and behavioral intention [65]. Attraction in involvement shows the highest predictive power on recommendation intention. Kyle and Mowen [19] discovered that an individual would enhance the motivation to participate in a leisure activity when considering a specific leisure activity with the value of stress relief and physical health promotion. In this case, enhancing the value of karate could promote karate participants’ intention to recommend it to others. Lifestyle centrality and self-performance also reveal a certain influence on recommendation intention. Accordingly, having karate participants regard karate as a part of life or assisting participants in achieving self-realization through karate would promote participants’ recommendation intention.

Karate participants’ perceived value appears to have remarkably positive effects on recommendation intention. Yang et al. [71] proved that sports participants’ perceived value would influence the subsequent behavioral intention. Perceived value is directly used for discussing sports participants in this study, and the result proves that perceived value would affect karate participants’ recommendation intention. Such a result conforms to the conclusion of Chuang et al. [72], Shih et al. [85], and Hu et al. [86] about perceived value, behavioral intention, and revisit intention. The higher perceived value of participants would strengthen the intention to recommend to others.

Physiological benefits in leisure benefits do not reveal a notable influence on recommendation intention, while overall leisure benefits show significantly positive effects on recommendation intention. The conclusion of remarkably positive effects of overall leisure benefits on recommendation intention conforms to Wang’s [77] research on the relationship between recreation benefits and after-visit behaviors as well as the relationship between leisure benefits and loyalty in the study of Pi et al. [78]. Nevertheless, physiological benefits do not appear to have notable effects on karate participants’ recommendation intention. Such a result seems to be different from general awareness. Originated from self-defense and fighting techniques, karate is a kind of martial art developed for cultivating body and spirit and exercise [6]. The factor in participating in martial art is generally considered the relationship with exercise; therefore, physiological benefits after participation should affect participants’ subsequent behavioral intention. The mean of participants’ physiological benefits in this study reached 4.498, the highest among the subdimensions of leisure benefits, showing that participants highly affirm the physiological benefits resulting from participating in karate. However, the result does not reveal a notable influence of physiological benefits on recommendation intention. It might be that participants, before participating in karate, have a certain awareness about physiological benefits after participation. The actual physiological benefits after participation are consistent with the awareness before participation so that the satisfaction with physiological benefits is not surprising. It therefore does not affect participants’ recommendation intention.

## 6. Conclusions

From the results in this study, managers in karate teaching institutes should utilize various opportunities and places for exercise in order to enhance the exchange capability of karate participants. Participants could be familiar from conversation with others through the activity so as to deepen karate participants’ lifestyle centrality in involvement and social benefits from leisure benefits, as well as enhance the recommendation ability and intention.

In this study, we used three variables (involvement, perceived value, and leisure benefits) to understand the effect of karate participants’ recommendation intention. Future studies may investigate the reason for karate participants’ participation and actual recommendation to others, or use the other variables such as personality traits, identity, etc. In addition, future investigators can use in-depth interviews with qualitative research methods to analyze and compare the difference.

## Figures and Tables

**Table 1:** Analysis of sample basic data.

Variable	Category	No.	%	Variable	Category	No.	%
Gender	male	266	72.1%	Personal monthly income	Without income	96	26%
	female	103	27.9%		below 20,000 NTD	50	13.6%
Age	15~18	30	8.1%		20,001~35,000 NTD	73	19.8%
	19~22	72	19.5%		35,001~50,000 NTD	56	15.2%
	23~30	88	23.8%		50,001~70,000 NTD	43	11.7%
	31~40	90	24.4%		above 70,001 NTD	51	13.8%
	41~50	55	14.9%	Karate participation years	occasional participation	27	7.3%
	51~60	21	5.7%		within 1 year	22	6%
	above 61	13	3.5%		1~3 years	64	17.3%
Marital status	single	221	59.9%		4~10 years	108	29.3%
	married without child	20	5.4%		11~20 years	71	19.2%
	married with child	110	29.8%		21~30 years	39	10.6%
	others	18	4.9%		over 31 years	38	10.3%
Educational attainment	under junior high schools	9	2.4%	Karate rank	grade 1~3 (brown belt)	45	12.2%
	senior high schools	68	18.4%		grade 4~9 (red, purple, blue, green, yellow, orange belt respectively)	35	9.5%
	colleges or universities	214	58%		beginning degree (black belt)	96	26%
	higher than graduate schools	78	21.1%		degree 2~3 (black belt)	125	33.9%
Occupation	students	116	31.4%		degree 4~5 (black belt)	51	13.8%
	government employees	39	10.6%		above degree 6 (black belt)	17	4.6%
	service industry	48	13%	Average times of weekly exercise participation in past three months	less than 1 time	106	28.7%
	manufacturing industry	29	7.9%		1~2 times	108	29.3%
	commercial industry	31	8.4%		3~4 times	81	22%
	housekeepers	7	1.9%		5~7 times	25	6.8%
	self-employed	29	7.9%		more than 7 times	49	13.3%
	others	70	19%	Other martial art sports participation seniority	without participating	155	42%
Place of residence	north	155	42%		occasional participation	94	25.5%
	central	136	36.9%		within 1 year	27	7.3%
	south	57	15.4%		1~3 years	36	9.8%
	east	8	2.2%		4~10 years	31	8.4%
	outlying islands	13	3.5%		11~20 years	15	4.1%
					21~30 years	8	2.2%
					more than 31 years	3	0.8%

**Table 2:** Analysis of research variable mean.

Variable	Dimension	Mean	SD
Involvement	Attraction	4.497	0.556
	Lifestyle centrality	3.642	0.817
	Self-performance	3.889	0.778
	Overall involvement	4.010	0.622
Perceived value	Money	4.146	0.794
	Time	4.371	0.684
	Spirit and energy	4.507	0.613
	Overall perceived value	4.341	0.605
Leisure benefits	Physiological benefits	4.498	0.517
	Psychological benefits	4.468	0.591
	Social benefits	3.887	0.742
	Overall leisure benefits	4.284	0.521
Recommendation intention	Informing advantages	4.201	0.775
	Recommending to relatives, friends, and colleagues	4.098	0.848
	Suggesting participation	4.276	0.830
	Sharing knowledge	4.431	0.727
	Overall recommendation intention	4.251	0.682

**Table 3:** Reliability and validity analysis of questionnaire.

Dimension	No. of Questions	Cronbach’s α
Involvement scale	15	0.917
Perceived value scale	3	0.828
Leisure benefits scale	12	0.904
Recommendation intention scale	4	0.878
Total scale	34	0.953

**Table 4:** Correlation analysis.

Variables	Involvement	Perceived Value	Leisure Benefits	Recommendation Intention
Involvement	1			
Perceived value	0.345 *	1		
Leisure benefits	0.267 **	0.338 *	1	
Recommendation intention	0.587 **	0.549 **	0.621**	1

*: *p <* 0.05, **: *p <* 0.01.

**Table 5:** Multiple regression analysis between subdimensions of involvement and recommendation intention.

Dependent Variable	Independent Variable	*β*	VIF	*R* ^2^	Adj. *R*^2^	*F*
Recommendation intention	Attraction	0.330 ***	1.919	0.357	0.352	67.510 ***
	Lifestyle centrality	0.247 ***	2.137			
	Self-performance	0.100 *	1.782			

*: *p* < 0.05; ***: *p <* 0.001.

**Table 6:** Regression analysis between perceived value and recommendation intention.

Dependent Variable	Independent Variable	*β*	*R* ^2^	Adj. *R*^2^	*F*
Recommendation intention	Perceived value	0.619 ***	0.302	0.300	158.668 ***

***: *p <* 0.001.

**Table 7:** Multiple regression analysis between subdimensions of leisure benefits and recommendation intention.

Dependent Variable	Independent Variable	*β*	VIF	*R* ^2^	Adj. *R*^2^	*F*
Recommendation intention	Physiological benefits	0.053	1.979	0.400	0.395	81.173 ***
	Psychological benefits	0.401 ***	2.259			
	Social benefits	0.311 ***	1.478			

***: *p* < 0.001.

**Table 8:** Test results of research hypotheses.

Research Hypothesis	Test Results
H1: Karate participants’ involvement positive affects the recommendation intention	Supported
H2: Karate participants’ perceived value positive affects the recommendation intention	Supported
H3: Karate participants’ leisure benefits positive affects the recommendation intention	Partially supported
